# Supplementary material for: High-Density Lipoprotein Particles and Their Relationship to Posttransplantation Diabetes Mellitus in Renal Transplant Recipients
Source: Biomolecules. 2020 Mar 21;10(3):481. doi: 10.3390/biom10030481 (PMC7175217; doi:10.3390/biom10030481)
Supplement: Supplementary file 1 [file biomolecules-10-00481-s001.pdf]

biomolecules

MDPI

## Supplementary Materials

**Table S1.** Spearman correlations between HDL paricles, LDL-P, and TRL-P

| Variables | Large HDL | Medium HDL | Small HDL | LDL-P | TRL-P |
|---|---|---|---|---|---|
| Large HDL | - | 0.089* | -0.236*** | -0.225*** | -0.225*** |
| Medium HDL | - | - | -0.392*** | -0.043 | -0.058 |
| Small HDL | - | - | - | 0.145** | 0.136** |
| LDL-P | - | - | - | - | 0.395*** |
| TRL-P | - | - | - | - | - |

*P<0.05;: **P<0.01; ***P<0.001

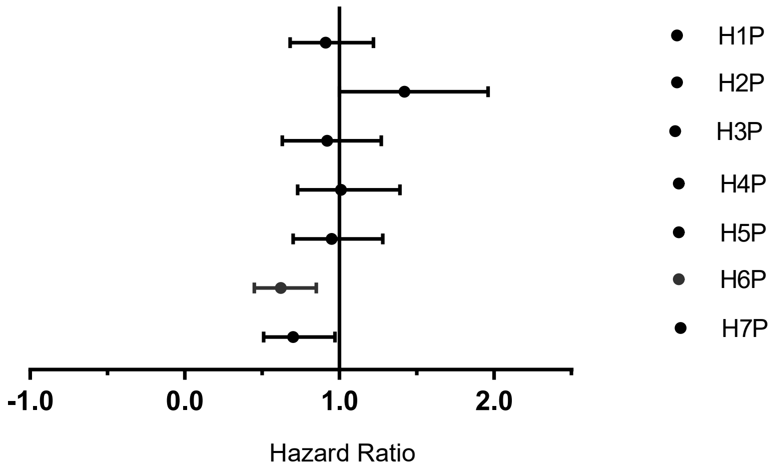


**Figure S1**. Association between Pasma HDL subspecies and risk of PTDM.Hazard ratio (95% CIs) per 1SD increase in each HDL subspeceis derived from Cox proportional hazards model for PTDM events (n=39 total events)

biomolecules

MDPI

**Table S2**. Association between large HDL subspecies and risk of PTDM in 351 RTRs

| Tertiles | 1 | 2 | 3 | | |
|---|---|---|---|---|---|
| H6P µmol/L | >1.2 | 0.4-1.1 | <0.4 | Per 1SD Log H6P | *P* value |
| Cases | 4 | 14 | 21 | 39 | |
| Crude analysis | 1.00 (ref) | 4.04 (1.33-12.29) | 6.15 (2.11-17.9) | 0.62 (0.45-0.85) | 0.003 |
| Model 1 | 1.00 (ref) | 4.77 (1.22-11.62) | 5.90 (1.98-17.57) | 0.64 (0.47-0.89) | 0.007 |
| Model 2 | 1.00 (ref) | 3.26 (1.03-10.28) | 5.69 (1.89-17.11) | 0.64 (0.45-0.90) | 0.010 |
| Model 3 | 1.00 (ref) | 3.80 (1.22-11.79) | 6.61 (2.20-19.84) | 0.60 (0.43-0.83) | 0.002 |
| Model 4 | 1.00 (ref) | 3.66 (1.16-11.50) | 6.12 (2.03-18.44) | 0.63 (0.45-0.88) | 0.006 |
| Model 5 | 1.00 (ref) | 3.44 (1.11-10.65) | 5.06 (1.66-15.41) | 0.71 (0.50-0.99) | 0.047 |
| Model 6 | 1.00 (ref) | 3.61 (1.15-11.28) | 5.39 (1.77-16.37) | 0.68 (0.49-0.95) | 0.024 |
| H7P µmol/L | >0.4 | 0.2-0.4 | <0.2 | Per 1SD Log H7P | *P* value |
| Cases | 5 | 20 | 14 | 39 | |
| Crude analysis | 1.00 (ref) | 3.53 (1.32-9.41) | 3.65 (1.31-10.14) | 0.70 (0.51-0.97) | 0.034 |
| Model 1 | 1.00 (ref) | 3.14 (1.17-8.46) | 3.18 (1.13-8.99) | 0.75 (0.53-1.06) | 0.10 |
| Model 2 | 1.00 (ref) | 2.89 (1.06-7.85) | 2.88 (1.00-8.26) | 0.76 (0.54-1.07) | 0.11 |
| Model 3 | 1.00 (ref) | 3.48 (1.29-9.41) | 3.07 (1.08-8.72) | 0.77 (0.55-1.08) | 0.12 |
| Model 4 | 1.00 (ref) | 3.09 (1.14-8.35) | 2.65 (0.92-7.60) | 0.82 (0.58-1.15) | 0.25 |
| Model 5 | 1.00 (ref) | 2.24 (0.82-6.10) | 2.13 (0.74-6.13) | 0.93 (0.65-1.33) | 0.69 |
| Model 6 | 1.00 (ref) | 3.19 (1.16-8.78) | 2.39 (0.80-7.10) | 0.90 (0.64-1.29) | 0.58 |

HRs (95% CIs) were derived from Cox proportional hazard models. Multivariable model 1 was adjusted for age, sex, and BMI. Model 2 adjusted for model 1 variables amd alcohol consumption, smoking, and physical activity; Model 3 adjusted for model 1 variables and treatment (lipid-lowering medication, anti-hypertensive medication, prednisolone dose, calcineurin inhibitors, and proliferation inhibitors); Model 4 adjusted for model 1 variables and eGFR, urinary albumin excretion, CMV infection, time after transplantation; Model 5 adjusted for model 1 variables and HbA1c; Model 6 adjusted for model 1 variables and systolic blood pressure, fasting blood glucose and triglycerides.
